# Cell-free production of integral membrane aspartic acid proteases reveals zinc-dependent methyltransferase activity of the *Pseudomonas aeruginosa* prepilin peptidase PilD

**DOI:** 10.1002/mbo3.51

**Published:** 2012-12-17

**Authors:** Khaled A Aly, Emily T Beebe, Chi H Chan, Michael A Goren, Carolina Sepúlveda, Shin-ichi Makino, Brian G Fox, Katrina T Forest

**Affiliations:** 1 Department of Bacteriology, University of Wisconsin-MadisonMadison, WI, 53706; 2 Department of Biochemistry, University of Wisconsin-MadisonMadison, WI, 53706; 3 Transmembrane Protein Center, University of Wisconsin-MadisonMadison, WI, 53706

**Keywords:** In vitro translation, liposome, *S*-adenosyl methionine, type II secretion, type IV pili

## Abstract

Integral membrane aspartic acid proteases are receiving growing recognition for their fundamental roles in cellular physiology of eukaryotes and prokaryotes, and may be medically important pharmaceutical targets. The Gram-negative *Pseudomonas aeruginosa* PilD and the archaeal *Methanococcus voltae* FlaK were synthesized in the presence of unilamellar liposomes in a cell-free translation system. Cosynthesis of PilD with its full-length substrate, PilA, or of FlaK with its full-length substrate, FlaB2, led to complete cleavage of the substrate signal peptides. Scaled-up synthesis of PilD, followed by solubilization in dodecyl-β-d-maltoside and chromatography, led to a pure enzyme that retained both of its known biochemical activities: cleavage of the PilA signal peptide and *S*-adenosyl methionine-dependent methylation of the mature pilin. X-ray fluorescence scans show for the first time that PilD is a zinc-binding protein. Zinc is required for the *N*-terminal methylation of the mature pilin, but not for signal peptide cleavage. Taken together, our work identifies the *P. aeruginosa* prepilin peptidase PilD as a zinc-dependent *N*-methyltransferase and provides a new platform for large-scale synthesis of PilD and other integral membrane proteases important for basic microbial physiology and virulence.

## Introduction

**Figure 1:**
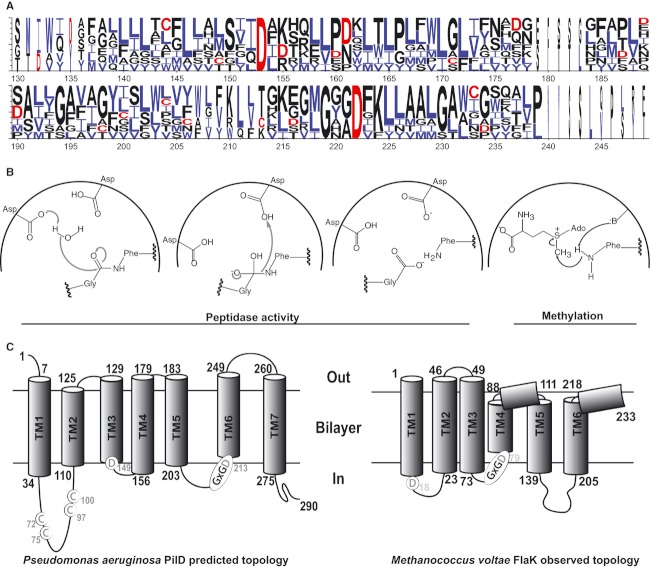
Sequence, mechanism, and membrane topology of integral membrane aspartic acid proteases (IMAAPs). (A) Local sequence alignment of eight prokaryotic IMAAP domains: *Pseudomonas aeruginosa* PilD (genbank accession number NP_253218), *Escherichia coli* K-12 GspO (NP_417794), *Neisseria gonorrhoeae* PilD (AAC43468), *Vibrio cholerea* TcpJ (AAK20796), *Xanthomonas campestris* XpsO (AAC43571), *Methanococcus voltae* FlaK (AAM34242), *P. aeruginosa* FppA (NP_252985), and *Sulfolobus solfataricus* PibD (DAA02293), with logo display from WebLogo3.1 (Crooks et al. 2004). (B) Schematic representation of the enzymatic mechanism of aspartic acid proteases and *S*-adenosyl methionine (SAM)-dependent methyltransferases. Two aspartic acid residues comprising the protease active site catalyze the deprotonation of a water molecule, resulting in the attack on the substrate carbonyl carbon at the target peptide bond and release of the signal peptide in a typical acid–base mechanism (Erez et al. 2009). In SAM-dependent methylation, the positively charged sulfonium group in SAM promotes an electrophilic character in the three surroundings carbon atoms, which allows the nucleophilic attack by an electron-rich heteroatom as N from phenylalanine, in an S_N_2 mechanism. (C) Predicted membrane topology of *P. aeruginosa* PilD and *M. voltae* FlaK (the latter confirmed by crystal structure; Hu et al. 2011). Two IMAAP-invariant aspartic acid residues (including the second within the GXGD motif) and four PilD-conserved cysteine residues are circled.

Integral membrane aspartic acid proteases (IMAAPs) represent a superfamily of proteins that exists in all three domains of life. In humans, IMAAPs play a catalytic role in the processing of β- and γ-secretase substrates and a defect in their enzymatic activity contributes to the development of Alzheimer's disease (Steiner and Haass 2000; Sastre et al. 2001; Weyand et al. 2010). In archaea, IMAAPs mediate signal peptide cleavage from preflagellins, prepilins, and solute-binding protein precursors, leading to assembly on the cell surface of archaella, type IV pili, and carbohydrate bindosomes, respectively (Bardy and Jarrell 2002, 2003; Albers et al. 2003; Albers and Meyer 2011; Jarrell and Albers 2012). IMAAPs are required for binding, processing, and transporting of DNA into competent cells of Gram-positive bacteria (Dubnau 1997). In Gram-negative bacteria, IMAAPs have been extensively studied in *Neisseria gonorrhoeae*, *Vibrio cholerae*, *Pseudomonas aeruginosa*, and others where they serve as the dedicated prepilin signal peptidase for maturation of type IV pilin subunits (Nunn and Lory 1991; Strom et al. 1993a,b; Freitag et al. 1995; LaPointe and Taylor 2000). Although IMAAP enzymatic activity is not inhibited by classical aspartic acid protease inhibitors (LaPointe and Taylor 2000), two invariant aspartic acid residues form the catalytic active site (Fig. 1A and B). The second of these is embedded in a GXGD motif, which provides a common moniker for this group of proteases. Both aspartic acids are near the boundary between the cytoplasm and inner leaflet of the membrane in the only known structure of an IMAAP, the recently published FlaK (Fig. 1C) (Hu et al. 2011).

In *P. aeruginosa*, IMAAPs are exemplified by the prepilin peptidase PilD. PilD removes a positively charged six amino acid *N*-terminal signal peptide of the prepilin PilA. PilA is a type IVa pilin (T4Pa), and thus has a signal peptide with glycine and phenylalanine residues at positions −1 and +1, respectively (Essex-Lopresti et al. 2005). On signal peptide cleavage, PilD additionally methylates the *N*-terminal phenylalanine of PilA prior to pilus assembly (Strom et al. 1993b). PilD similarly processes the major and minor pseudopilins that constitute the *P. aeruginosa* type II secretion (T2S) pseudopilus (Nunn and Lory 1993; Pepe and Lory 1998). Whereas signal peptide cleavage of PilA is a prerequisite for pilus assembly, the *N*-terminal methylation of the processed pilin is not; the role of the methylation is unknown (Pepe and Lory 1998). PilD and other IMAAPs from the bacterial T4Pa and T2S systems contain four cysteine residues (C72, C75, C97, and C100 in PilD) (Strom et al. 1993a) in a domain predicted to fall on the cytoplasmic side of the inner membrane (Fig. 1C) based on alkaline phosphatase fusions and membrane topology predictions (Strom and Lory 1987; Dupuy et al. 1991). In addition to the cysteines, a glycine and a basic amino acid adjacent to the third cysteine contribute to the methylation reaction in vivo and in vitro (Pepe and Lory 1998). Thus, the two conserved aspartic acid residues of PilD constitute the active site required for signal peptide cleavage of prepilin, whereas the four-cysteine domain plays a less fully explored role in the subsequent *N*-terminal methylation of the mature pilin. The precise locations of these two enzymatic activities with respect to the lipid bilayer are unclear, but are likely to be near the interface of the cytoplasm and the inner leaflet of the inner membrane based on (i) the structure of FlaK (Hu et al. 2011), (ii) the prediction that the mature *N*-terminus of the PilA subunit floats within the bilayer rather than being exposed in the cytoplasm (Lemkul and Bevan 2011), and (iii) the cytoplasmic source of the predicted methyl donor *S*-adenosyl methionine (SAM) (Strom and Lory 1987).

In order to advance understanding of this important class of enzymes, we undertook a cell-free synthesis of representative prokaryotic IMAAPs and their substrates. A large-scale production and purification pipeline was successfully developed for PilD. We demonstrated a role of the cytoplasmic four-cysteine domain in metal binding as a prerequisite for methylation.

## Experimental Procedures

### Bacteria and plasmid constructions

Strains and plasmids used in this study are described in Table 1. Cultures of *Escherichia coli* JM109 for cloning experiments were grown at 37°C in Luria–Bertani medium (1% peptone, 0.5% yeast extract, 0.5% NaCl), and DNA manipulations followed standard procedures (Maniatis et al. 1982). Antibiotics were added for plasmid propagation (kanamycin, 75 μg/mL; ampicillin, 100 μg/mL). *Pseudomonas aeruginosa pilD* and *pilA* or *Methanococcus voltae flaK* and *flaB2* were polymerase chain reaction (PCR) amplified from chromosomal DNA libraries prepared using the Wizard® Genomic DNA Purification Kit (Promega, Madison, WI) and the oligonucleotide primers listed in Table 1. Various DNA fragments were directly ligated into the pCR®II-Blunt-TOPO® vector (Invitrogen, Grand Island, NY), followed by DNA sequence verification (UW Biotechnology Center). Fragments were excised using *Sgf*I and *Pme*I followed by subsequent ligation into pEU-C-His Flexi (Sevova et al. 2010) precut with *Sgf*I and *EcoICR*I, resulting in *pFlexipilD*, *pFlexipilA*, *pFlexiflaK*, and *pFlexiflaB2*, respectively. The stop codons of *pilD* and *flaK* were removed during oligonucleotide design, resulting in C-terminally 6x-His-tagged proteins by virtue of the histidine tag encoded by the pEU-C-His Flexi plasmid.

**Table 1:** Primers, strains, and plasmids

Primer/strain/plasmid	Genotype/description	Source/reference
Primers		
*pilD*-5′	5′-GGTTGCGATCGCC*ATG*CCCCTCCTCGACTACCTG-3′	This study
*pilD*-3′	5′-GTGTGTTTAAACTTTGAATCCGGCGAATTGCAGATAGG-3′	This study
*pilA*-5′	5′-GGTTGCGATCGCC*ATG*AAAGCTCAAAAAGGCTTTACCT-3′	This study
*pilA*-3′	5′-GTGTGTTTAAAC*TCA*GTTATCACAACCTTTCGGAGTGAACAT-3′	This study
*flaK*-5′	5′-GGTTGCGATCGCC*ATG*GCATATACAATAGGACTTTTGGGA-3′	This study
*flaK*-3′	5′-GTGTGTTTAAACCAATGGCAATATAATACTAATTATTTTA-3′	This study
*flaB2*-5′	5′-GGTTGCGATCGCC*ATG*AAAATAAAAGAATTCATGAGTAACAAAAA-3′	This study
*flaB2*-3′	5′-GTGTGTTTAAAC*CTA*TTGTAATTGAACTACTTTTGAATCGTTTAA-3′	This study
Strains		
*Escherichia coli* JM109	*endA1 gyr96 thi hsdR71 supE44 recA1 relA1* Δ*(lac-proAB) (F' traD36 proAB*^*+*^ *lacI*^*q*^ *lacZ*Δ*M15)*	Yanisch-Perron et al. (1985)
Plasmids		
pEU-C-His Flexi	Amp^r^, SP6 promoter expression vector	Sevova et al. (2010)
pFlexi*pilD*	pEU-C-His Flexi 870 bp *pilD* gene from *Pseudomona aeruginosa* strain PAK	This study
pFlexi*pilA*	pEU-C-His Flexi 450 bp *pilA* gene from *P. aeruginosa* strain PAK	This study
pFlexi*flaK*	pEU-C-His Flexi 699 bp *flaK* gene from *Methanococcus voltae*	This study
pFlexi*flaB2*	pEU-C-His Flexi 651 bp *flaB2* gene from *M. voltae*	This study

Sgf1 restriction enzyme cleavage sites in 5′ (forward) primers and PmeI sites in 3′ (reverse) primers are underlined, native start and stop codons are italicized.

### Preparation of unilamellar liposomes

Liposomes were prepared as previously described (Goren and Fox 2008). Briefly, liposomes were prepared from soybean tissue extract (Avanti Polar Lipids, Alabaster, AL). The lipid powder was dissolved in chloroform and dried for 30 min under vacuum after removal of the organic solvent by evaporation under a stream of N_2_ gas. The dried lipid film was rehydrated at a concentration of 15 mg/mL with 25 mmol/L HEPES (*N*-[2-hydroxyethyl]piperazine-*N*-2[ethanesulphonic acid]), pH 7.5, containing 100 mmol/L NaCl. The lipid solution was vortexed for 5 min and subjected to three freeze–thaw cycles. An Avanti mini-extruder was used to form unilamellar liposomes by 11 passes through a 100-nm track-etch polycarbonate membrane (Nucleopore, Pleasanton, CA). The liposomes were stored at −80°C.

### Transcription reactions

Small-scale transcription reactions followed recently published protocols (Goren et al. 2009). The total volume of a transcription mixture was 50 μL. Reaction components included 4 μg of purified plasmid DNA, 80 mmol/L HEPES, pH 7.5, 16 mmol/L magnesium acetate, 2 mmol/L spermidine, 10 mmol/L dithiothreitol (DTT), 2.5 mmol/L of each nucleotide triphosphate (ATP, UTP, GTP, and CTP), 25 U of RNasin (Promega), and 30 U of SP6 RNA polymerase (Promega). Reactions were incubated for 4 h at 37°C. Large-scale transcription reactions had minor differences in reagent concentrations (Beebe et al. 2011).

### Cell-free translation

The small-scale translation mixtures of IMAAPs and/or substrates followed the protocol of Goren and Fox (Goren et al. 2009). The total reaction volume of 50 μL contained 30 mmol/L HEPES, pH 7.8, 100 mmol/L potassium acetate, 2.7 mmol/L magnesium acetate, 1.2 mmol/L ATP, 0.25 mmol/L GTP, 16 mmol/L creatine phosphate, 0.4 mmol/L spermidine, 0.3 mmol/L of each amino acid, 0.7 mg/mL of creatine kinase, 24 U of RNasin, and 60 μg of liposomes. Wheat germ extract (15 μL) was added from a concentrated commercial preparation (CellFree Sciences, Yokohama, Japan). The purified mRNA pellet from the transcription reaction was dissolved in this translation mixture and placed into a 12-kDa MWCO dialysis cup (Cosmo Bio, Tokyo, Japan). The cup was suspended in a buffer reservoir containing the above reagents except creatine kinase, RNasin, liposomes, and wheat germ extract. After overnight incubation at 26°C, resulting proteoliposomes were pelleted by microcentrifugation for 5 min at maximum speed, followed by three washes with 50 μL of 25 mmol/L HEPES, pH 7.5.

### Scale-up of PilA and PilD synthesis

Large-scale (4–8 mL) translation reactions for PilA and PilD were carried out on a Protemist 10/100 robot, under conditions similar to those of the small-scale reaction, but without added liposomes (Beebe et al. 2011).

The 4 mL PilA translation was aliquoted and flash-frozen in liquid nitrogen and stored at −80°C. For peptidase activity tests, aliquots of PilA were thawed, centrifuged at 20,000*g* for 5 min, and the pellet washed in one of two assay buffers (10 mmol/L BisTris, pH 7.0, 100 mmol/L NaCl, 2 mmol/L DTT [Fig. 3B], or 5 mmol/L MES pH 6.0, 50 mmol/L NaCl [Fig. 5]). The PilA pellet was solubilized in assay buffer containing 0.1% dodecyl-β-d-maltoside (DDM) for 1 h at 25°C.

The frozen whole 8-mL translation reaction of PilD was solubilized in 9.5 mL of 50 mmol/L NaH_2_PO_4_, pH 8.0, 25 mmol/L imidazole, 300 mmol/L NaCl, 2 mmol/L DTT supplemented with 1% DDM for 1 h at 25°C. Insoluble material was removed by centrifugation at 20,000*g* for 5 min, and the soluble fraction was used for protein purification.

### PilD purification

One percent DDM-solubilized 6x-His-tagged PilD from an 8-mL translation reaction was applied to a 1-mL HisTrap HP column on an AktaPrime purification system (GE Healthcare, Piscataway, NJ) equilibrated in 50 mmol/L NaH_2_PO_4_, pH 8.0, 50 mmol/L imidazole, 300 mmol/L NaCl, 2 mmol/L DTT containing 0.1% DDM, followed by washing in the same buffer, and eluting with 50 mmol/L NaH_2_PO_4_, pH 8.0, 500 mmol/L imidazole, 300 mmol/L NaCl, 2 mmol/L DTT, and 0.05% DDM. Peak fractions were pooled and concentrated by centrifugation at 4°C in Amicon Ultra-50K 4-mL spin concentrators. Concentrated protein was subjected to buffer exchange against 10 mmol/L Bis-Tris, pH 7.0, 100 mmol/L NaCl, 0.02% NaN_3_, 2 mmol/L DTT, and 0.05% DDM. Affinity-purified PilD was applied to a Superdex-75 10/300 GL size exclusion column (GE Healthcare) and retention times were compared to a standard calibration curve to determine apparent molecular weight. The final sample was concentrated to ∼5.5 mg/mL in 5 mmol/L 2-(*N*-morpholino)ethanesulfonic acid (MES), pH 6.0, 0.3 mmol/L Tris(2-carboxyethyl)phosphine (TCEP), 50 mmol/L NaCl, and 0.2% DDM, using a 50-kDa MWCO spin concentrator. This final detergent concentration, which is higher than that of the buffer alone due to the fact that some detergent is concentrated along with the protein, was estimated by electrospray ionization–time of flight (ESI-TOF) mass spectrometry, coupled to a hydrophobic interaction-high-performance liquid chromatography column (Phenomonex Luna 3 μm, 2.00 × 150 mm) run with an ∼2–90% acetonitrile gradient, generated using 10 mmol/L ammonium acetate pH 7.0 (buffer A) and 95% acetonitrile in water (buffer B) at a flow rate of 200 μL/min. There was no apparent loss of enzymatic activity after two rounds of freeze–thawing of PilD.

### Peptidase activity, ^14^C-SAM preparation and radioactive methylation experiments

For the peptidase activity tests, purified 6x-His-PilD was incubated with cell-free-synthesized PilA in assay buffer with 0.1% DDM (described above) in an approximate molar ratio of 1:5 for 1 h at 37°C. Lower ratios sometimes led to incomplete cleavage, indicating purified PilD acts as a *bona fide* enzyme, although we note that we have not calculated a formal specific activity and do not mean to claim that all of the PilD molecules are enzymatically active. An equal volume of protein sample buffer was added to stop the reaction prior to loading 15% SDS-PAGE (sodium dodecyl sulfate polyacrylamide) gels, which were visualized with Coomassie staining, Western blotting or silver staining.

For methylation experiments, [methyl-^14^C] SAM was generated using adenosine triphosphate (ATP) and l-methionine [methyl-^14^C] (55 mCi/mmol American Radiolabeled Chemicals) with *Saccharomyces cerevisiae* SAM synthetase (SAM2) as previously described (Park et al. 1996). The SAM synthetase reaction was performed in 100 mmol/L Tris pH 7.1, 1 mmol/L TCEP, 100 mmol/L KCl, 26 mmol/L MgCl_2_, 60 mmol/L ATP, l-methionine (20 mmol/L [18.2 mmol/L unlabeled + 1.8 mmol/L methyl-^14^C]), and 10 μmol/L enzyme. Next, the synthetase reaction was incubated for 4 h at 30°C prior to use in radioactive methylation experiments. PilD methylation experiments were performed as described for the peptidase assay in the presence of 30% v/v of the SAM synthetase reaction mixture as the source of SAM. Where indicated, 10 mmol/L ethylenediaminetetraacetic acid (EDTA) and/or 5 mmol/L ZnCl_2_ were added.

### X-ray fluorescence scan

Purified 6x-His-tagged PilD described above was mixed with an equal volume of precipitating agent (0.2 mol/L (NH_4_)_2_SO_4_, 25% polyethylene glycol 3350, 0.1 mol/L Bis-Tris pH 4–6). This solution was mixed with 1/10th volume of 50 mmol/L methyl-β-cyclodextrin, and suspended from a glass cover slip over a reservoir of precipitating agent. This experiment led to amorphous gels that did not diffract, but were highly concentrated PilD. These were scooped into crystallization loops (Hampton Research, Aliso Viejo, CA) followed by cryoprotection in low viscosity cryoprotection oil-1 cryoprotection oil (MiTeGen). X-ray fluorescence scans were carried out at the GM/CA-CAT beamline, Advanced Photon Source (APS), Argonne National Laboratory (ANL), Chicago, IL.

### Multiple sequence alignment and membrane topology predictions

Protein sequences of various IMAAPs were prepared in a FASTA format followed by local alignments using the COBALT multiple sequence alignment tool (Papadopoulos and Agarwala 2007). Plotting of the membrane topology predictions was based on the results obtained from the Hidden Markov Model for predicting transmembrane helices in protein sequences (TMHMM) server, Center for Biological Sequence Analysis (CBS), Technical University of Denmark.

### Production of polyclonal pilin antisera

Preimmune serum from three rabbits was screened against a whole-cell lysate of *P. aeruginosa* strain 103 (PA103), and a rabbit with low background signal was chosen for initial and booster immunizations with purified pili from this strain. Pili were sheared from plate-grown PA103 cells, and purified through three rounds of precipitation in Tris-buffered saline alternated with resuspension in high pH, low osmolarity buffer as described (Craig et al. 2003). A primary injection with this preparation was followed by two booster injections with pilin further subjected to SDS-PAGE and elution from the gel slice containing pilin. The antibody signal was checked after each of these injections, and after the third check the rabbit was sacrificed and the antiserum collected. Antibody production was carried out at the UW-Madison Medical School.

### SDS-PAGE and Western blotting

SDS-PAGE was conducted according to Laemmli (Laemmli 1970). Stain-free visualization of proteins takes advantage of tryptophan fluorescence (Kazmin et al. 2002) and is done using a Criterion Stain Free Imager (BioRad, Hercules, CA). Western blotting was performed following standard protocols with the above-described PA103 PilA antisera (1:10,000 dilution) or with FlaB2-specific chicken antibodies (1:5000) (Bayley and Jarrell 1999) and appropriate secondary antibodies. PA103 pilin and PAK pilin share 67% sequence identity.

## Results

### In vitro synthesized PilD and FlaK are active peptidases

An alignment of the core region of bacterial and archaeal IMAAPs highlights the two aspartic acid residues that constitute the active site (Fig. 1A) and are expected to catalyze peptidase activity by a classic acid–base mechanism requiring a water molecule (Fig. 1B). Using the hidden Markov Model TMHMM server (Sonnhammer et al. 1998), we predicted the membrane topology of both the *P. aeruginosa* PilD and the *M. voltae* FlaK (Fig. 1C), and find that the latter is supported by the recent crystal structure of this protein (Hu et al. 2011). Both proteins are characterized by the presence of an aqueous loop, but in the *P. aeruginosa* PilD the loop contains the four-cysteine domain that is not found in the *M. voltae* FlaK possible cytoplasmic loop (Fig. 1C).

**Figure 2:**
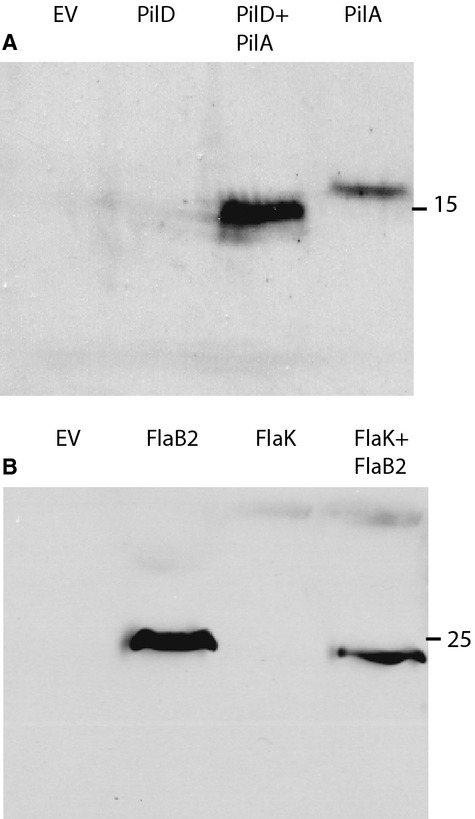
Cell-free syntheses yield *Pseudomonas aeruginosa* PilD and *Methanococcus voltae* FlaK active against cognate substrates. The final pellet from a small-scale translation reaction was resuspended in 25 mmol/L HEPES, pH 6.5 then used in these activity assays. (A) PilD activity: pellets of the translation reactions of the empty vector (EV); PilD; PilD + PilA cotranslation and PilA were separated by SDS-PAGE, followed by Western blot analysis using PilA-specific antiserum. (B) FlaK activity: pellets of the translation reactions of the EV; FlaB2 substrate, FlaK enzyme, and FlaK + FlaB2 cotranslation were separated by SDS-PAGE, followed by Western blot analysis using FlaB2-specific antibodies.

Using cell-free synthesis, C-terminally 6x-His-tagged PilD and FlaK were independently transcribed and subsequently translated in the presence of unilamellar liposomes in a wheat germ-based cell-free system (Goren and Fox 2008) (Fig. 2A and B). To test whether in vitro synthesized PilD and FlaK are biochemically active, each protein was cosynthesized with its full-length substrate. PilD cosynthesized with its partner substrate PilA was biochemically active as assessed by a size shift between the full-length and processed PilA products (Fig. 2A). PilD also cleaved the major pseudopilin from the type II secretion system, XcpT, but had no activity against the noncognate substrate FlaK (data not shown). A similar result was obtained when the archaeal preflagellin peptidase FlaK was cosynthesized with its full-length substrate, FlaB2 (Fig. 2B). These results indicate that the multipass IMAAPs and their single-pass integral membrane protein substrates are successfully produced in the in vitro transcription and translation reactions, and the in vitro synthesized PilD and FlaK are biochemically active proteases that retain their target specificities.

### C-terminally 6x-His-tagged PilD remains biochemically active upon purification

**Figure 3:**
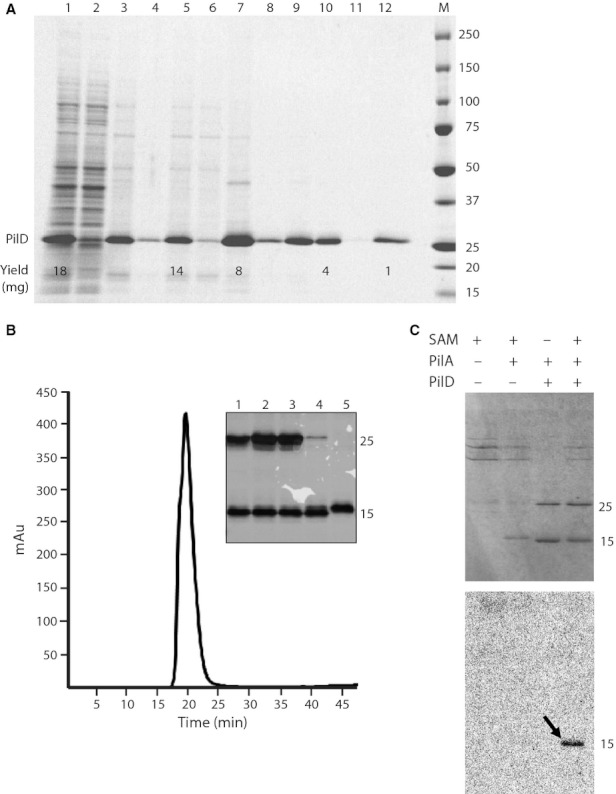
Purification, peptidase, and methyltransferase activities of PilD. (A) Large-scale cell-free synthesis and two-step purification. Lane 1: total translation reaction, 2: translation reaction soluble fraction (not used in purification); 3: total sample after solubilization in 1% dodecyl-β-d-maltoside (DDM); 4: pellet after solubilization; 5: soluble fraction in 1% DDM (applied to immobilized metal-affinity chromatography [IMAC] column); 6: flow-through from IMAC; 7: elution from IMAC; 8: elution fractions concentrated, pellet; 9: elution fractions concentrated, soluble (applied to gel filtration); 10: pooled gel filtration peak fractions; 11: concentrated gel filtration sample, pellet; 12: concentrated gel filtration sample, soluble. Approximate yields are based on tryptophan fluorescence from the PilD band (Kazmin et al. 2002). (B) Gel filtration chromatogram indicates monodispersity of the C-terminally 6x-His-tagged PilD (independent purification from a). Inset: Silver-stained gel showing peptidase activity of PilD. In each case an equal amount of PilA protein (prepared as described in the Experimental Procedures and reclarified by centrifugation at 20,000*g* for 5 min) was incubated with an equal volume of the indicated PilD sample or buffer. Lane 1: pooled gel filtration peak fractions; 2: peak fractions after concentration; 3: concentrated gel filtration sample, soluble; 4: concentrated gel filtration sample, pellet; and 5: no PilD added. (C) Peptidase and methyltransferase activities of purified PilD. Upper panel: Coomassie stained 15% SDS-PAGE gel. Lower panel: Autoradiograph of the same gel. Lanes are labeled with reaction components. *S*-adenosyl methionine (SAM) synthetase reaction mixture (high-molecular-weight [MW] bands) serves as the source of freshly synthesized radiolabeled ^14^C-SAM.

While biochemically active, the levels of FlaK synthesized by cell-free approach were considerably lower than PilD. Therefore, we decided to scale up the production of PilD for further analysis. To this end, 4 mL of the cell-free synthesis reaction was prepared for the production of PilD. The overall expression level was ∼2 mg/mL of translation reaction (Fig. 3a). Unexpectedly, we observed that in the absence of unilamellar liposomes, endogenous wheat germ extract lipids provided an alternative lipid source and supported synthesis of the active integral membrane protease PilD (Ostlund et al. 2003). The proteoliposome pellet of the cell-free reaction (Fig. 3A, lane 3) was solubilized in 1% dodecyl-β-d-maltoside (DDM) (Fig. 3A, lane 5), followed by immobilized metal affinity chromatography (IMAC) of the solubilized protein over Ni–NTA resin (Fig. 3A, lane 7). Elution of PilD in the presence of DDM concentrations ranging from 0.5% to 0.025% did not affect the yield upon concentration (Fig. 3A, lane 9), and this highly enriched sample retained approximately 50% of the initial translation product (Fig. 3A, lane 9). Further purification using size exclusion chromatography (Fig. 3A, lane 10, unconcentrated; lane 12, concentrated; and Fig. 3B) led to a monodisperse sample with a molecular mass of ∼68 kDa. Although not conclusive, this size is consistent with monomeric PilD (30.9 kDa) complexed with detergent. After this two-step purification, PilD retained its peptidase activity when incubated with independently synthesized full-length PilA (Fig. 3B, inset).

### Purified PilD methylates the mature pilin

In order to assess if *in vitro* synthesized PilD retains its second enzymatic function, a radioactive methylation assay was developed in which purified PilD was incubated with independently synthesized full-length PilA in the presence of the radioactive methyl donor ^14^C-SAM. ^14^C-SAM was synthesized immediately before use in a reaction containing partially purified, bacterially expressed SAM synthetase with ATP and radiolabeled methionine. PilD methylates the mature pilin in the presence of radioactive SAM (Fig. 3C). *N*-terminal sequence analysis was used on a parallel nonradiolabeled sample to demonstrate the first amino acid was uniquely modified as expected (Fig. S1).

### Zinc is bound to PilD and is required for pilin methylation

**Figure 4:**
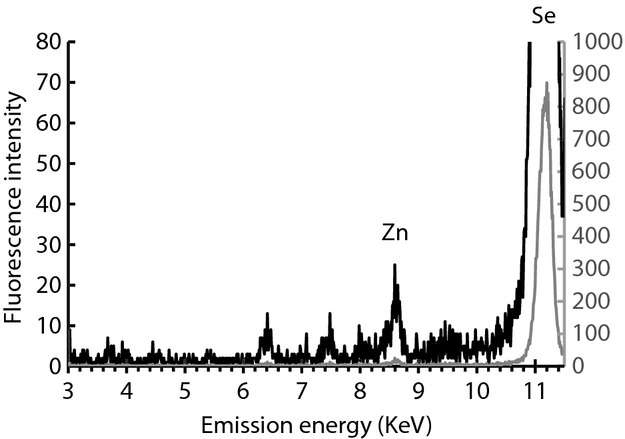
X-ray fluorescence scan of Se-Met-labeled PilD. Sample was prepared as described in the Experimental Procedures, with selenomethionine in place of methionine in the amino acid mix. In this X-ray fluorescence scan for elemental analysis, excitation at 12,660 eV allowed observation of the strong Se emission peak from the 12 methionines in PilD as well as the Zn emission peak. Identical data are plotted on two scales to highlight the Zn signal.

The four cysteines at positions 72, 75, 97, and 100 in PilD (Fig. 1C) have some hallmarks of general metal-binding motifs (CxxC-x_*n*_-CxxC), therefore we sought to determine whether PilD binds metal. X-ray fluorescence is a sensitive and highly specific method for elemental analysis of possible bound metals from small noncrystalline samples (Qin et al. 2011). We suspended and vitrified a highly concentrated jelly-like sample of detergent-solubilized PilD in the loop of a crystallization pin, followed by an X-ray fluorescence scan at the GM/CA beamline at the Advanced Photon Source. Using PilD synthesized with selenomethionine rather than methionine provided the internal control of selenium fluorescence in an emission scan carried out using excitation at the Se K edge (Fig. 4). In addition to the expected Se fluorescence at ∼11.3 KeV, a second weaker peak was detected at ∼8.6 KeV indicating that PilD is bound to zinc (Fig. 4).

**Figure 5:**
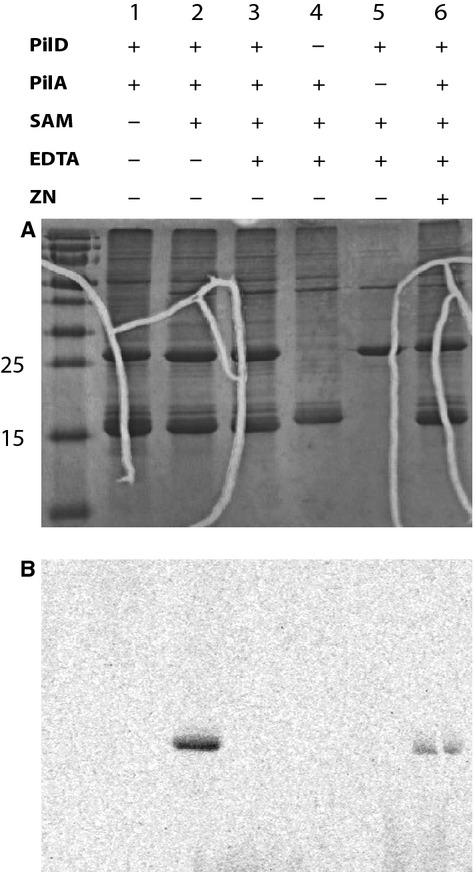
Removal of zinc prevents methylation, but not proteolysis by PilD. (A) Purified PilD cleaves the signal peptide from PilA (pelleted, washed, and resuspended after translation, but not further purified) during a 1-h reaction at 37°C in the absence (lane 1) or presence (lane 2) of *S*-adenosyl methionine (SAM), whether PilD was exposed to 10 mmol/L EDTA (lane 3) or a buffer control (lane 2) for 1 h at room temperature prior to the reaction. PilA alone is not cleaved, as indicated by its higher molecular weight (lane 4). Freshly synthesized radiolabeled ^14^C-SAM was added to indicated reactions in the form of a SAM synthetase reaction mix (SAM synthetase appears as the high-molecular-weight [MW] bands in this Coomassie-stained 15% SDS-PAGE gel). (B) Autoradiograph of the same gel. SAM is required (lane 2), whereas EDTA inhibits PilD-dependent methylation of PilA (lane 4). PilD alone is not radiolabeled (lane 5). Methylation activity was restored to the EDTA-treated enzyme by addition of zinc to a 5 mmol/L final concentration at the onset of the reaction (lane 6, note the esthetically unpleasing crack serves as a fiducial for the radiolabeled PilA in the upper and lower gels).

The fact that PilD binds zinc begs the question what the role of zinc is in PilD function. Therefore, we sequestered zinc by incubating PilD with 10 mmol/L EDTA prior to enzymatic assay. Whereas zinc removal did not inhibit signal peptide cleavage of PilA (Fig. 5A, lane 3), it abolished pilin methylation (Fig. 5B, lane 3). After EDTA treatment of the PilD/PilA reaction mixture, the addition of 5 mmol/L ZnCl_2_ partially restored methylation activity (Fig. 5B, lane 6).

## Discussion

One bottleneck in biochemical and structural characterization of integral membrane proteins is their low yield from cell-based methods for overproduction and purification. In this study, we have successfully applied a relatively simple cell-free approach for the production of two full-length members of the IMAAP superfamily, the Gram-negative *P. aeruginosa* prepilin peptidase PilD, and the archaeal *M. voltae* preflagellin peptidase FlaK, as well as their full-length substrates, PilA and FlaB2, respectively (Goren and Fox 2008; Goren et al. 2009; Makino et al. 2010; Sevova et al. 2010; Beebe et al. 2011). Our results demonstrate that the wheat germ cell-free approach is suitable for the production of biochemically active IMAAPs.

This study documents, for the first time, metal binding by PilD. The presence of four cysteine residues in the predicted cytoplasmic loop of PilD in a CXXC-X_21_-CXXC arrangement triggered our speculation that this motif might bind a metal, a finding supported by X-ray fluorescence. We note that we have not formally demonstrated that PilD within *Pseudomonas aeruginosa* cells is bound to zinc, but the only alternatives are (i) no metal, which could be a regulatory state during which methylation will not occur, or (ii) a different metal. The latter case is unlikely: in four-cysteine metal-binding sites in the protein data bank, only Zn^2+^, Fe^2+^, or Co^2+^ are found (Dokmanic et al. 2008) and in PilD the two sets of paired cysteines with a long intervening linker are hallmarks of Zn-finger-type sites rather than iron–sulfur clusters, which generally have closer sequence spacing (Aitken 1999; Tus et al. 2012).

The bound zinc in PilD is functionally important, and moreover helps demarcate requirements for the two enzymatic activities of PilD, as its removal blocks the methyltransferase activity without affecting signal peptide cleavage. PilD variants with amino acid substitutions in each of the four cysteines have been previously studied and were shown to affect pilin methylation (Strom et al. 1993a), but not protease activity (LaPointe and Taylor 2000). Effects on peptidase activity were also originally reported, but were likely the result of nonspecific structural changes. Moreover, the four-cysteine domain is not found in all IMAAPs and thus cannot be expected to be an inherent requirement for peptidase activity. Our suspicion is that this zinc-finger-like motif plays a structural role in the folding and stability of the cytoplasmic domain of PilD and/or is necessary for substrate recognition and binding of either pilin or SAM. SAM, in particular, is found in the cytoplasm and must be shepherded into an active site within the membrane.

Structural homology is strong among soluble SAM-dependent methyltransferases despite a lack of sequence conservation (Martin and McMillan 2002), but are these soluble structures predictive for the membrane-embedded PilD? A germane recent study of a unique helical integral membrane *O*-methyltransferase revealed a cytoplasmic SAM-binding pocket at the base of a tunnel leading into the hydrophobic interior of the membrane (Yang et al. 2011). We also considered the possibility that the pairs of cysteines might function in formation of the disulfide bond in folded pilin, either directly or by serving as a substitute for the activity of DsbB to reduce the periplasmic disulfide bond isomerase DsbA (Kadokura and Beckwith 2010). However, the mismatch between the predicted cytoplasmic location of the four-cysteine domain and the periplasmic requirement for this function make this scenario unlikely. A complex function in regulation of pilus production in response to redox changes, similar to redox regulation of the zinc-binding chaperone Hsp33 (Jakob et al. 2000), is similarly intriguing although to date not described for T4P.

All of the proteins in this study were synthesized in vitro using eukaryotic wheat germ extract with soy-derived liposomes or endogenous lipids. Retention of enzymatic activity suggests these provide interactions with the proteins that mirror those of their phosopholipid counterparts in prokaryotic biological membranes. The cell-free approach is thus an attractive and accessible method for high-yield production of functional full-length IMAAPs, opening a new avenue for biochemical, mechanistic, and structural studies.

## Supporting Information

**Figure S1.** SAM-dependent methylation of PilA is specific for Phe1.
