# Supplementary material for: Cell-free production of integral membrane aspartic acid proteases reveals zinc-dependent methyltransferase activity of the Pseudomonas aeruginosa prepilin peptidase PilD
Source: Microbiologyopen. 2012 Dec 17;2(1):94–104. doi: 10.1002/mbo3.51 (PMC3584216; doi:10.1002/mbo3.51)
Supplement: Supplementary file 1 [file mbo30002-0094-SD1.pdf]

Cell-free Production of Integral Membrane Aspartic Acid Proteases Reveals Zinc-dependent Methyl Transferase Activity of the *Pseudomonas aeruginosa* Prepilin Peptidase PilD

Khaled Ahmed Aly, Emily T. Beebe, Chi Ho Chan, Michael A. Goren, Carolina Sepúlveda, Shin-ichi Makino, Brian G. Fox and Katrina T. Forest

Supplementary Figure 1. SAM-dependent methylation of PilA is specific for Phe1.

Chromatograms from N-terminal protein sequencing show SAM-dependent modification of the first residue, phenylalanine. Separate suspensions of PilD and PilA translation reactions were mixed together at final concentrations of 0.2% DDM, 100 mM HEPES-NaOH (pH 7.5), 1 mM DTT, 0.3 mg/mL PilD, and 0.9 mg/mL PilA (molar ratio of PilD:PilA=1:6.3) in the presence or absence of 80 μM SAM, and incubated at 37° C for 2 h.

A reference chromatogram of amino acid derivative standards (10 pmol each) is shown on the top, in which two peaks other than 20 amino acids, diphenylthiourea (dptu) and diphenylurea (dpu), are by-products of Edman degradation. N-methylphenylalanine (NmF) was detected with a retention time around 19 min, well-separated from the other amino acid derivatives, which is consistent to an increased hydrophobicity over phenylalanine (Strom *et al.*, 1993). The amount of each amino acid residue detected in each cycle is as follows: 31 pmol, F, residue 1 (control); 26 pmol, T, residue 2 (control); 22 pmol, T, residue 2 (+SAM).

Sequencing experiments were carried out at the Protein Facility of the Iowa State University. We are grateful to Joel Nott for assistance.

Reference

Strom, M. S., D. N. Nunn & S. Lory, (1993) A single bifunctional enzyme, PilD, catalyzes cleavage and N-methylation of proteins belonging to the type IV pilin family. *Proc Natl Acad Sci U S A* **90**: 2404-2408.

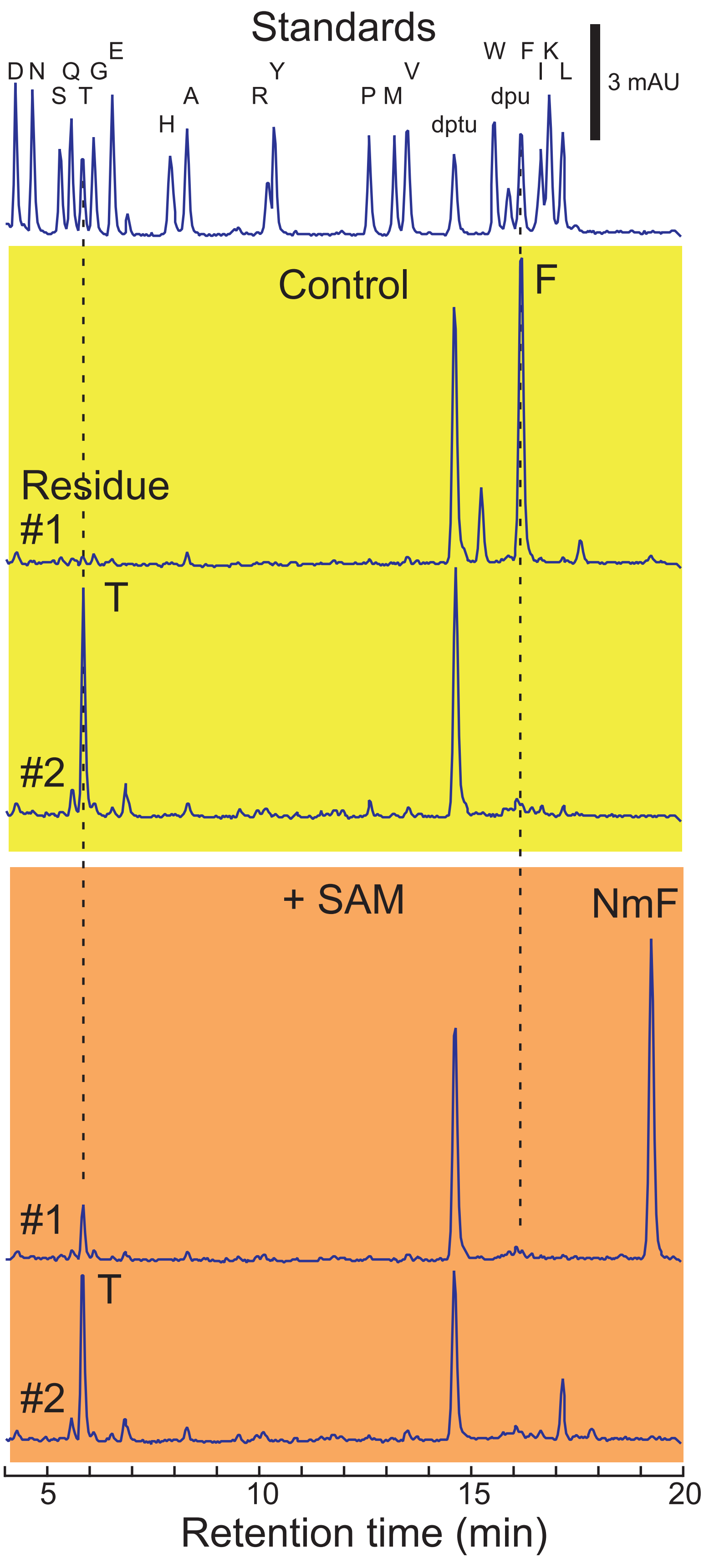

Aly *et al.*, Suppl. Fig. 1
